# The protective effects of pericyte-derived microvesicles on vascular endothelial functions via CTGF delivery in sepsis

**DOI:** 10.1186/s12964-021-00795-y

**Published:** 2021-11-16

**Authors:** Henan Zhou, Danyang Zheng, Hongchen Wang, Yue Wu, Xiaoyong Peng, Qinghui Li, Tao Li, Liangming Liu

**Affiliations:** 1 State Key Laboratory of Trauma, Burns and Combined Injury, Shock and Transfusion Department, Army Medical Center of PLA, Daping Hospital, Army Medical University, No.10th Daping Changjiang Road, Chongqing, 400038 China; 2 Intensive Care Unit, General Hospital of Central Theater Command, Wuhan, 430064 China

**Keywords:** Pericyte, Microvesicles, Sepsis, CTGF

## Abstract

**Background:**

It is well known that sepsis is a prevalent severe disease caused by infection and the treatment strategies are limited. Recently pericyte-derived microvesicles (PMVs) were confirmed to be therapeutic in many diseases, whether PMVs can protect vascular endothelial cell (VEC) injury is unknown.

**Methods:**

Pericytes were extracted from the retina of newly weaned rats, and PMVs were collected after starvation and characterized by flow-cytometry and transmission electron microscopy. First, the effect of PMVs on pulmonary vascular function in septic rats was measured via intravenous administration with HE staining, immunofluorescence, and Elisa analysis. Then, PMVs were co-incubated with VECs in the presence of lipopolysaccharide (LPS), and observed the protective effect of PMVs on VECs. Next, the proteomic analysis and further Gene Ontology (GO) enrichment analysis were performed to analyze the therapeutic mechanism of PMVs, and the angiogenesis-related protein CTGF was highly expressed in PMVs. Finally, by CTGF upregulation and downregulation in PMV, the role of PMV-carried CTGF was investigated.

**Results:**

PMVs restored the proliferation and angiogenesis ability of pulmonary VECs, and alleviated pulmonary vascular leakage in septic rats and LPS-stimulated VECs. Further study showed that PMVs delivered CTGF to VECs, and subsequently activated ERK1/2, and increased the phosphorylation of STAT3, thereby improving the function of VECs. The further study found CD44 mediated the absorption and internalization of PMVs to VECs, the anti-CD44 antibody inhibited the protective effect of PMVs.

**Conclusions:**

PMVs may delivery CTGF to VECs, and promote the proliferation and angiogenesis ability by activating the CTGF-ERK1/2-STAT3 axis, thereby protecting pulmonary vascular function in sepsis. The therapeutic effect of PMVs was highly related to CD44-mediated absorption.

**Video Abstract**

**Supplementary Information:**

The online version contains supplementary material available at 10.1186/s12964-021-00795-y.

## Introduction

Sepsis is a life-threatening disease caused by the body's severe response to infection causing a high incidence and mortality rate [1–3]. Vascular endothelial cells (VECs) are widely distributed in the internal walls of microvessels and large blood vessels, and consist of the barrier between blood and tissues, which are essential for vascular homeostasis and organ function [4–6]. When sepsis occurs, VECs are exposed to the outburst of inflammatory factors and toxins in the circulation, and inflammatory pathways such as NF-κB, RhoA/ROCK, MLCK-MLC are activated, and vasoconstriction and barrier function are impaired [7–9]. Meanwhile, the production of growth factors is decreased and the proliferation ability of VECs is inhibited, resulting in the dysfunction of the self-repair of vessels [6–10]. Since the treatment of vascular function mainly consists of vasopressors, injection of growth factors, and glucocorticoids, the effect is limited [6, 9]. As a result, it is an urgent need to find new treatment strategies to restore the function of VECs after sepsis.

Pericytes (PCs) are vascular multifunctional cells embedded in the basement membrane of vessels and extend processes both along and around microvessels and capillaries [11, 12]. Pericytes are found to regulate capillary reactivity in response to cell depolarization or inflammatory stimulation, due to their physiological characteristics [11–14]. Meanwhile, PCs can secrete pro-contractile and cytoskeletal-related proteins, which make PCs have the function of regulating blood flow and maintaining metabolic balance [15]. In addition, PCs have immune and secretory functions, participating in exogenous coagulation reactions and tissue regeneration [14, 15]. Moreover, several studies have found that PCs have multipotent stem cell potential, and can differentiate into macrophages, smooth muscle cells, etc. [16, 17], thus PCs could participate in embryonic development and disease regulation. Our previous studies found that exogenous infusion of PCs significantly reduced the hyperpermeability of vascular endothelial cells and mesenteric microvascular leakage in sepsis, suggesting that PCs had a protective effect on vascular functions in sepsis, however, the mechanism still needs further study.

Microvesicles (MVs) are membrane-enclosed nanoscale particles released from cells that contain proteins, lipids, RNA, and DNA [18–20]. MVs are able to deliver bioactive information between cells through the extracellular space and have been implicated in many physiological and pathological processes over the past decade [18–20]. The potential application of stem cell-derived MVs attracted increasing attention, as MVs have a similar therapeutic effect in contrast to mother stem cells. For example, mesenchymal stem cell (MSC)-derived MVs (MMVs) from human were as effective as the parent stem cells in increasing alveolar fluid clearance and improving airway and hemodynamic parameters [21]. On the other hand, MVs are natural membrane vesicles secreted, thus they are proved to have lower immunogenicity and toxicity, and are more efficiently absorbed by target cells than other synthetic delivery vehicles, such as liposomes, micelles, dendrimers and nanocapsules [22]. It has been proved that MMVs may promote the proliferation of osteoblasts through regulating transcription activity and expression level of genes [23, 24]. There were studies to report pericytes-derived microvesicles (PMVs) from human cerebrovascular sources may carry neuroprotective factors, including VEGF, BDNF, and PLGF, to reduce nerve cell damage [25–27]. Therefore, we put forward the hypothesis that PMVs might deliver therapeutic molecules to VECs to improve vascular function after sepsis. Hence, septic rat model and VECs were used to investigate the therapeutic effect and mechanism of PMVs on vascular functions in sepsis.

## Materials and methods

### Ethical approval

In the present study, all experiments followed the Guide for Care and Use of Laboratory Animals by America (National Institute of Health) and approved by Army Medical University’s Ethics Committee and Research Council and Animal Care and Use Committee (Number. DHEC-2012-069).

### Animals’ preparation and sepsis model

Male and female Sprague–Dawley (SD) rats (with a weight range of 195 ± 10 g) were purchased from the Animal Research Center of the Daping Hospital (Army Medical Center). Then sepsis model was performed by cecal ligation and puncture (CLP) procedure as described [28, 29]. In brief, laparotomy was performed under anesthesia with pentobarbital sodium solution (45 mg/kg intraperitoneal), the cecum of rats was gently exposed, the feces were concentrated at the end of the cecum, then the surgical silk was used to ligate the end of the cecum. Afterward, rectangular needle was used to perforate the bling-end of the cecum, and the exposed intestine was placed back to abdominal cavity, then the abdomen was closed by interrupted suture. Finally, each of the rats was injected with 5 mL of normal saline intraperitoneally, and they were subsequently fed with water until use.

### Cell culture and reagents

Pericytes (PCs) were obtained from the retina of weanling SD rats [30]. The eyeballs were removed immediately after general anesthesia, and placed in PBS containing 1% penicillin/streptomycin. Afterward, the eyeball was separated from sclera and pigment epithelium using scissors and tweezers. Next, it was gently placed in the dissection buffer. The retina was snipped and digested with 2 mg/mL type I collagenase, and then DMEM containing 20% FBS was added to stop the digestion. After filtration and centrifugation, the mixed medium was cultured in 5 mL pericyte specific medium (ScienCell, America) in the presence of corresponding pericyte growth supplement (ScienCell, America) and antibiotics (ScienCell, America).

Vascular endothelial cells (VECs) were obtained from the left lung of rats as described [31]. In brief, thoracotomy was performed under anesthesia with pentobarbital sodium solution (45 mg/kg intraperitoneal). Later the pulmonary vein in the left lung was exposed gently and subsequently took out, and the vein was washed for 4 times and cut to pieces, then the vein pieces were placed in the culture flask and cultured (DMEM-F12 medium, 10% serum (Gibco, America).

Several studies have pointed out that, endotoxemia could be used to model the acute inflammatory response associated with sepsis and LPS was the major Gram negative bacteria [32, 33]. As a result, LPS was used as a stimulation in vivo*.*

LPS (055:B5) was purchased from Sigma (America). CCK8 Detection Kit was purchased from Dojindo (Japan), and TNFα Elisa Kit was purchased from Abcam (America). ERK1/2 agonist and ERK1/2 inhibitor were purchased from Selleck (America). STAT3 agonist was purchased from MedChemExpress (America). Anti-CD44 blocking antibody was purchased from Abcam (America).

### Harvest of MVs

MVs were harvested from cultured PCs supernatant [34]. Before the MV harvest, PCs were starved by serum-free basal medium for 24 h, then the supernatant was collected in sterile centrifuge tubes. The supernatant was processed as follows: 500 g for 20 min (4 °C, discard the precipitation), then 2000 g for 25 min (4 °C, discard the precipitation), then 20,000 g for 80 min (4 °C, discard the supernatant). The MV pellet was washed by PBS and stored at − 80 °C until subsequent analysis [35].

### Flow-cytometry (FCM)

PCs and PMVs were subjected to high sensitivity imaging flow-cytometry Amnis Image Stream MK II (ISX) (Amnis, America) and further analyzed by INSPIRE (v1.4.0). In brief, samples were stained with CD146 (1:100, BD, America), α-SMA (1:80, BD, America), CD140b (1:50, BD, America), NG (1:100, BD, America) and Annexin V (1:50, BD, America) for 30 min in the dark (RT). Annexin V binding buffer (BD, America) was used along with Annexin V antibody. Standard beads were added into MV samples (0.2 μm, 0.5 μm, 0.8 μm, Bangs laboratories, America) for the gate set and quantification of MVs.

### Transmission electron microscopy (TEM)

TEM of MVs were measured as described [34]. In brief, after fixing with 2.5% glutaraldehyde buffer (4 °C) for 24 h, the samples were washed 3 times by 0.1 M PBS. Then MVs were post-fixed by 0.1% OsO4 for 80 min (4 °C) and dehydrated in a graded ethanol series. Next, MVs were embedded in TAAB 812 and cut into 100 nm sections, then were observed by JEM 1400 (JEOL Instruments, Japan).

### Hematoxylin and Eosin (HE) staining

Rats were sacrificed gently, and the left lung was flushed with PBS formaldehyde-fixed, and finally it was embedded by paraffin and then cut into sections. After staining by HE, the sections were observed by microscopy (Leica, Germany).

### Immunofluorescence (IF) of lung

FITC-BSA (9 mg/kg) (Sigma, America) was injected into anesthetized rats through the jugular vein. After being flushed with PBS, the left lung was embedded in Tissue-Tek O.C.T. Compound (Sakura, America) at − 20 °C. When the compound was frozen, it was cut into sections by freezing microtome (Leica, Germany), and the sections were stained with DAPI (Abcam, America) and observed by confocal microscopy (Leica, Germany) [34, 36].

### Transendothelial electrical resistance (TER) and FITC-BSA leakage of VECs

TER and BSA leakage of VECs were detected as previously described [33]. In brief, VECs were seeded on the upper layer of Transwell (6-well, 0.4 μm, Coring, America), and VECs were added with different treatments when cells grew to full confluence (LPS: 2 μg/ml, PMV: 2 × 10^6^/ml). TER of VECs was assessed immediately after the treatment by oltohmmetre (World Precision Inc, America), and the value of TER was recorded every 30 min. For the assessment of BSA leakage, FITC-BSA (10 μg/ml) was added into VECs immediately after the treatment, and 200 μl supernatant of the lower insert of Transwell was collected and measured every 10 min, and 200 μl DMEM-F12 medium was supplemented into the upper insert to keep the balance of the culture system in Transwell.

### Angiogenesis measurement

Matrigel Matrix (Corning, America) was thawed at 4 °C and subsequently added in the µ-slides (ibidi, Germany) gently, then the µ-slides were incubated at 37 °C for 1 h. Afterward, VECs were seeded on the matrix and incubated with LPS and MVs according to the experiment design for 12 h. Then the µ-slides were labeled with calcein-AM (Thermo, America) and observed by Leica SP5 laser confocal microscopy (Germany) [37]. The tube length of angiogenesis analysis was further measured by imageJ software (v1.8.0).

### Western blot

Total protein mass was extracted by RIPA buffer from either tissues or cells, and SDS-PAGE and PVDF membrane was used. After being incubated with antibodies, the membrane was observed by Odyssey Clx (Li-Cor, America). PCNA (Proliferating Cell Nuclear Antigen), AKT, p38-MAPK, JNK and ERK1/2 were purchased from CST (America), CTGF and β-actin were purchased from Abcam (America).

### Migration of VECs

6-well plate (Corning, America) was used to detect the migration of VECs. Firstly, VECs were seeded at the concentration of 1 × 10^5^ per well and cultured to full confluence. Then the medium was change to DMEM-F12, and a sterile pipette was used to draw a straight line in the middle of the well. Afterward, VECs were washed and cultured with different treatments, and were observed by microscopy (Leica, Germany) at 0 h and 24 h.

### Pericyte transfection and harvest of modified PMVs

PCs were transfected with CTGF-shRNA adenovirus, CTGF-overexpressing adenovirus, and CTGF-mock adenovirus (Genechem Technology, China) according to experiments design. Briefly, corresponding adenovirus was co-incubated with PCs in the presence of Opti-MEM when cells were 70–80% confluence for 24 h (MOI = 80), then the medium was changed to exosome-free DMEM-F12 for another 24 h, and modified PMVs were harvested from the supernatant [29]. The target sequence of CTGF-shRNA was as follows:

Forward Strand: 5′-GATCCCTACCGACTGGAAGACACATTTCTCGAGAAATGTGTCTTCCAGTCGGTATTTTTGGAT-3′, Reverse Strand: 5-AGCTATCCAAAAATACCGACTGGAAGACACATTTCTCGAGAAATGTGTCTTCCAGTCGGTAGG-3′.

### LC–MS/MS analysis

PMVs were harvested as described (PC-MV1, PC-MV2, PC-MV3), and smooth muscle cell-derived microvesicles (SMVs) were used as control group (SMC-MV1, SMC-MV2, SMC-MV3). MV samples were analyzed by Applied Protein Technology Biolaboratory (China) by Q Exactive mass spectrometer (Thermo Scientific, America). Briefly, samples were resuspended in SDT buffer A (100 mM Tris–HCl, 4%SDS, 1 mM DTT, and pH was adjusted to 7.6) and quantified by BCA (Dojindo, Japan). Afterward, 200 μg protein was incorporated into SDT buffer B (30 μl, 150 mM Tris–HCl, 4% SDS, 100 mM DTT, and pH was adjusted to 8.0), then DTT and the detergent were removed by UA buffer (150 mM Tris–HCl,8 M Urea, pH was adjusted to 8.0) using ultrafiltration (Microcon units, 10 kD). Afterward, samples were added with 100 μl iodoacetamide (100 mM IAA in UA buffer) and incubated in darkness (0.5 h), and digested by trypsin (4 μg, Sigma, America) in 25 mM NH_4_HCO_3_ buffer overnight (RT), and these digested peptides were collected in a filtrate. Then peptides were desalted on C18 (Empore™ SPE Cartridges C18, 2.8 ml, bed I.D. 7 mm, Sigma, America), concentrated by vacuum centrifugation and reconstituted in 0.1%formic acid [38].

The purified peptides were loaded on the reverse phase trap column (Thermo Scientific, America), which was connected to the C18-reversed phase analytical column (Thermo Scientific, America) in 1‰ formic acid buffer A, then samples were separated by gradient buffer B (1‰ formic acid and 84% acetonitrile), with a flow rate of 300 nl/min. Then the purified peptides were analyzed by mass spectrometry and the data were collected by a data-dependent method dynamically choosing precursors ions from the scan range of 300–1800 m/z, and the parameters were as follows: 3e6 automatic gain control target; 10 ms maximum inject time; 40.0 s dynamic exclusion duration; a resolution of 70,000 at m/z 200 survey scans; 2 m/z isolation width; 30 eV normalized collision energy; peptide recognition mode [38].

### Database search and bioinformatic analysis

These MS/MS raw data were searched and analyzed by MaxQuant software (v1.5.3.09) for identification and quantitation information. Trypsin was used as cleavage enzyme, and the max missed cleavages were set to 2. Fixed modification was Carbamidomethyl (C), and variable modification was Oxidation (M), and the mass tolerance for precursor ions for First search and Main search was 20 ppm and 6 ppm, respectively. False discovery rate (FDR) was set to ≤ 0.01, and peptides for quantification were set as Use razor and unique peptides, and time window was 2 min. The differentially expressed (DE) proteins were identified with absolute fold change > 1.5 and *p* value < 0.05 [38].

The sequences of the DE proteins were searched and analyzed by the NCBI BLAST + client software (v2.2.10) and InterProScan (v5.0), and Gene Ontology (GO) annotation were mapped by BLAST2GO and plotted by R scripts. Then differentially expressed proteins were blasted against the Kyoto Encyclopedia of Genes and Genomes (KEGG) database (http://geneontology.org/) and mapped to pathways in KEGG. Proteomic enrichment analysis was performed based on the Fisher’ exact test, and *p* value < 0.05 was considered significant [38].

### Statistical analysis

In the present study, experiment data were analyzed by SPSS (v20.0, IBM, America). All the data were repeated at least 3 independent experiments, and results of one representative experiment were shown. Data were presented as mean ± SD, and difference among different groups were assessed by one-way ANOVA test, and values of *p* < 0.05 were considered significant.

## Results

### Characterization of PCs and PMVs

**Fig. 1:**
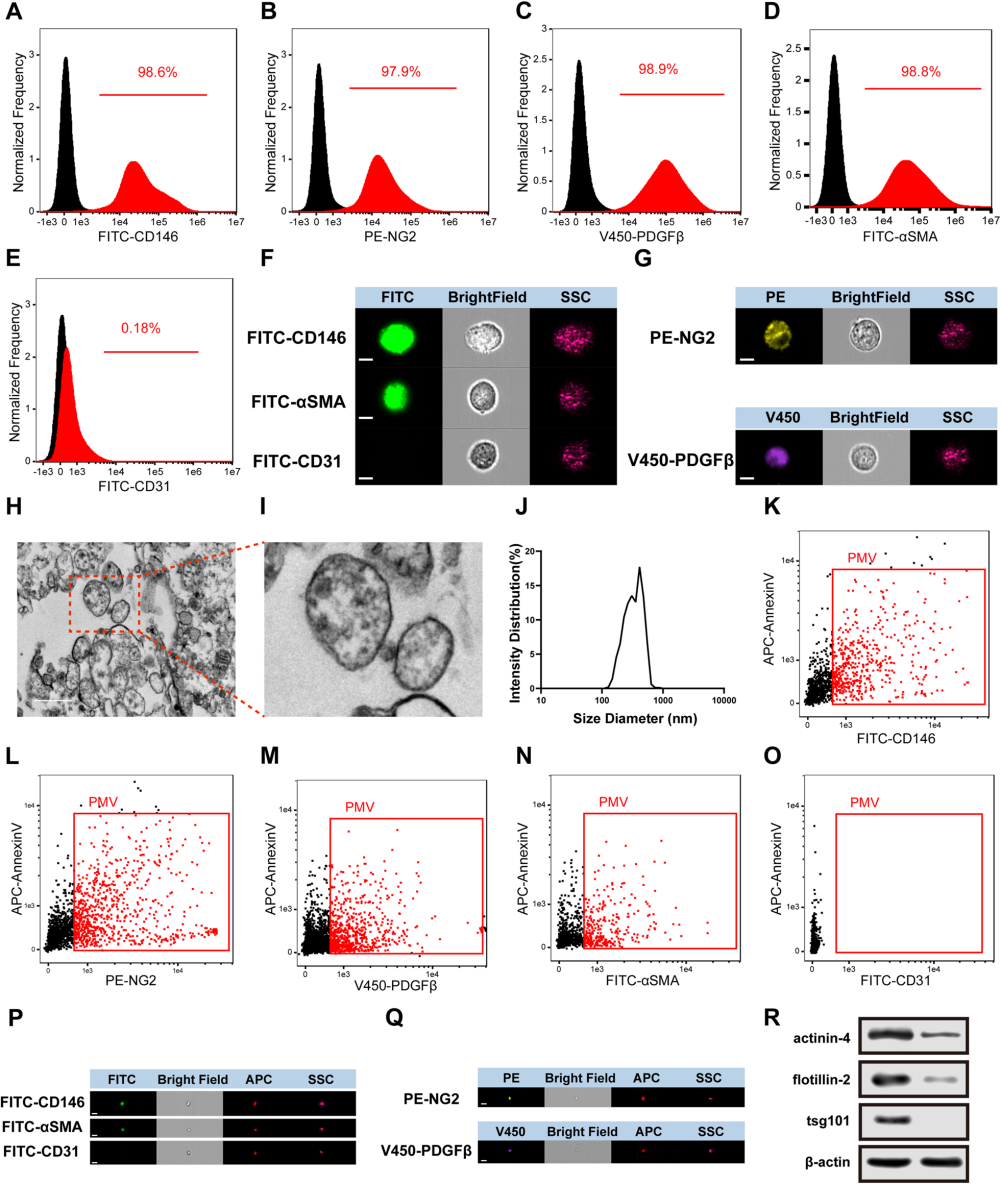
Characterization of PCs and PMVs. **A**–**E** Imaging flow-cytometry analysis of PCs, CD146, NG2, PDGFβ, and αSMA are the positive markers of PCs, CD31 is the negative marker of PCs. **F**, **G** Representative images of different markers for PCs, analyzed by imaging flow-cytometry (bar = 5 μm). **H**, **I** Representative images of PMVs, analyzed by transmission electron microscopy (bar = 500 nm). **J** Dynamic light scattering analysis of PMVs, which indicates the diameter distribution of PMVs (n = 3). **K**–**O** Imaging flow-cytometry analysis of PMVs. **P**, **Q** Representative images of different surface markers for PMVs, analyzed by imaging flow-cytometry (bar = 1 μm). **R** Western blot analysis of different markers in PMVs

PCs were harvested from the retina of weanling rats and were identified with specific surface markers by imaging flow-cytometry. The results showed that PCs were positive for markers α-SMA, PDGFR-β, and NG2, but negative for endothelial marker CD31, which confirmed PCs were pure (Fig. 1A–E). At the same time, imaging flow-cytometry presented the exact image of each marker of PCs (Fig. 1F, G), which further confirmed the purity of PCs.

In order to investigate the therapeutic effects of PMVs, PMVs were purified from the supernatant of PCs by ultracentrifugation and measured by electron microscopy firstly. The results showed that PMVs exhibited spherical morphology with apparent bimolecular membrane structure, and the size ranged from 100 to 1000 nm (Fig. 1H, I). Dynamic light scattering showed that the size of PMVs was from 142 to 892 nm (Fig. 1J). Imaging flow-cytometry showed that PMVs expressed PC marker NG2, PDGFR-β, and α-SMA, as well as MV marker Annexin V, and the endothelial marker CD31 were negative (Fig. 1K–Q). Besides, according to recent studies, a few proteins were used to distinguish MVs and exosomes, including tsg101, flotillin-2 and actinin-4 [35, 39–41]. The results showed that flotillin-2 and actinin-4 were enriched in PMVs and tsg101 was almost absent, which confirmed the purity of PMVs (Fig. 1R).

### PMVs protected lung tissue and improve pulmonary function in septic rats

To investigate the protective effects of PMVs on vascular function in sepsis, we administered PMVs to CLP rats (2 × 10^7^ MVs per rat, at 1 h after CLP) through the tail vein, and the vascular leakage was measured at 24 h after sepsis. Smooth muscle cells (SMCs) are one type of terminal cells differentiated from PCs and do not have stemness, as a result, SMC-derived MVs (SMVs) were used as a control group to investigate the therapeutic effects of PMVs. Hence, the same doses of SMVs were injected into CLP rats at 1 h after surgery, and the same volume of PBS (300 μL) was injected into normal and CLP rats at the same time.

**Fig. 2:**
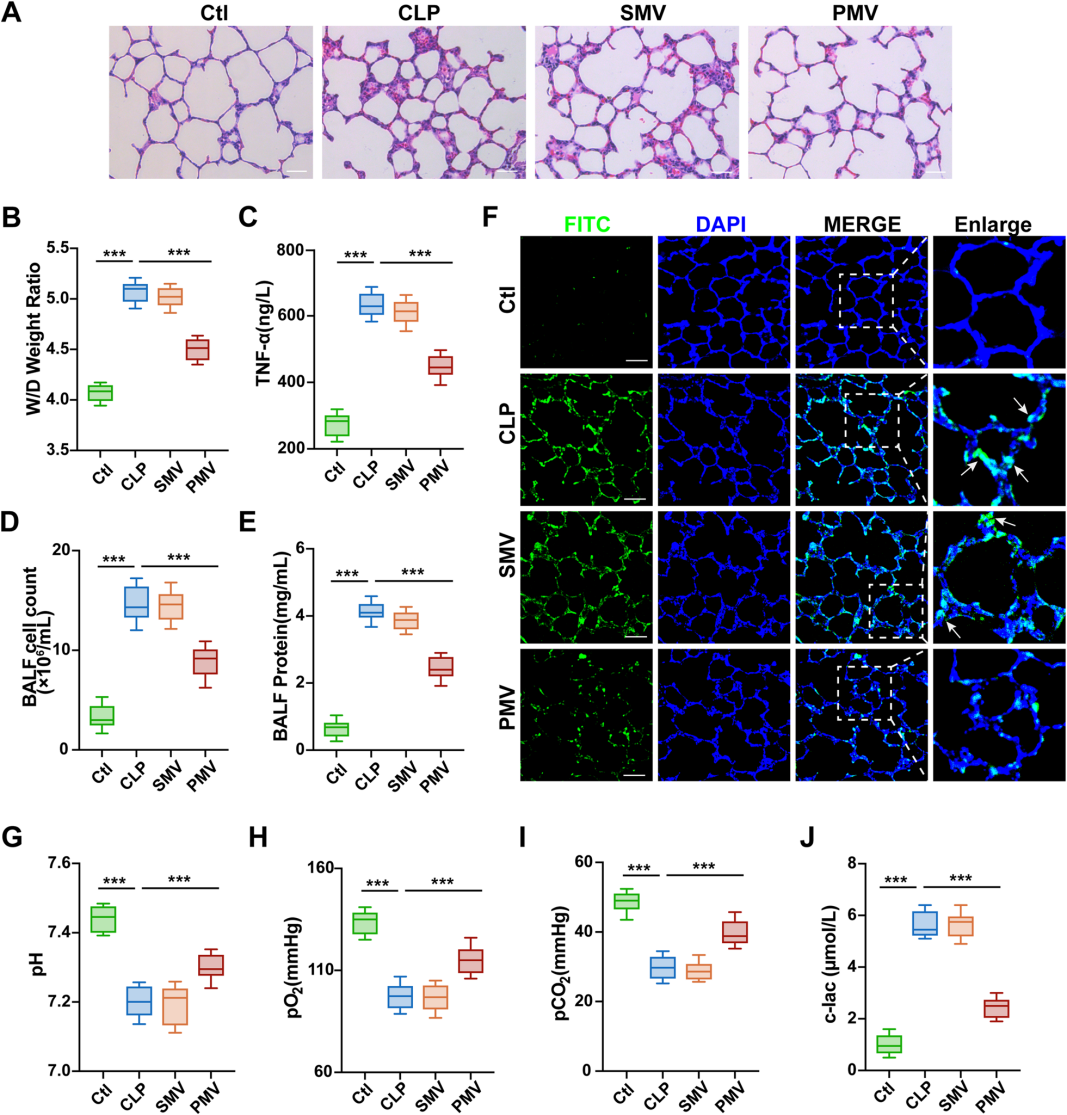
Protective effect of PMVs on pulmonary function in septic rats. **A** HE staining of the lung tissue, the representative images are shown (n = 5, bar = 40 μm). **B** Wet weight to Dry weight ratio of lung in rats (n = 8). **C** Serum TNF-α level in rats (n = 8). **D** Cell count in BALF (n = 8). **E** Protein concentration in bronchoalveolar lavage fluid (BALF) (n = 8). **F** Laser confocal microscopy analysis of FITC-BSA leakage in lung tissue, the representative images are shown (n = 5, bar = 50 μm). **G**–**J** Blood gas analysis, including pH, pO_2_, pCO_2_, cLac (n = 8). Ctl: normal group; CLP: sepsis group; SMV: SMV-treated sepsis group; PMV: PMV-treated sepsis group. ***P < 0.001

HE staining revealed that lung tissue was damaged after sepsis, featured with destroyed alveoli and thickened alveoli septum, while PMV treatment significantly alleviated the injury (Fig. 2A). Next, the lung water content was evaluated by the wet/dry weight ratio, compared to control group, the ratio raised significantly after sepsis. SMVs treatment did not affect the wet/dry weight ratio in contrast to the CLP group, and PMVs treatment decreased the ratio (Fig. 2B). The results showed that sepsis caused increased expression of TNF-α in blood, which was distinctly reduced by PMVs administration (Fig. 2C) as compared to SMVs group. And then, cell counts and protein concentration in bronchoalveolar lavage fluid (BALF) were measured, which represented the level of pulmonary vascular leakage induced by inflammation. The results showed that cell counts and protein concentration in BALF significantly increased after sepsis, and PMVs treatment improved the cell counts and protein concentration in BALF (Fig. 2D, E). Furthermore, the pulmonary vascular leakage to FITC-BSA was measured with confocal microscopy as a representative. It appeared that the vascular leakage of FITC-BSA was obviously increased after sepsis (Fig. 2F). The administration for SMVs did not decrease the pulmonary vascular permeability (*P* > 0.05), while PMVs could effectively alleviate the leakage. Additionally, significant changes in blood gas values were observed after sepsis, the levels of pH, pO2, pCO2 were each significantly decreased, whereas the lactate level was increased. The administration of PMVs could significantly improve blood gas indexes while SMVs had little effect, suggesting that PMV notably improved the pulmonary function after sepsis (Fig. 2G–J). Collectively, those data suggest that PMV treatment may alleviate pulmonary vascular leakage and improve lung function after sepsis.

### PMVs protected the function of LPS-stimulated VECs

**Fig. 3:**
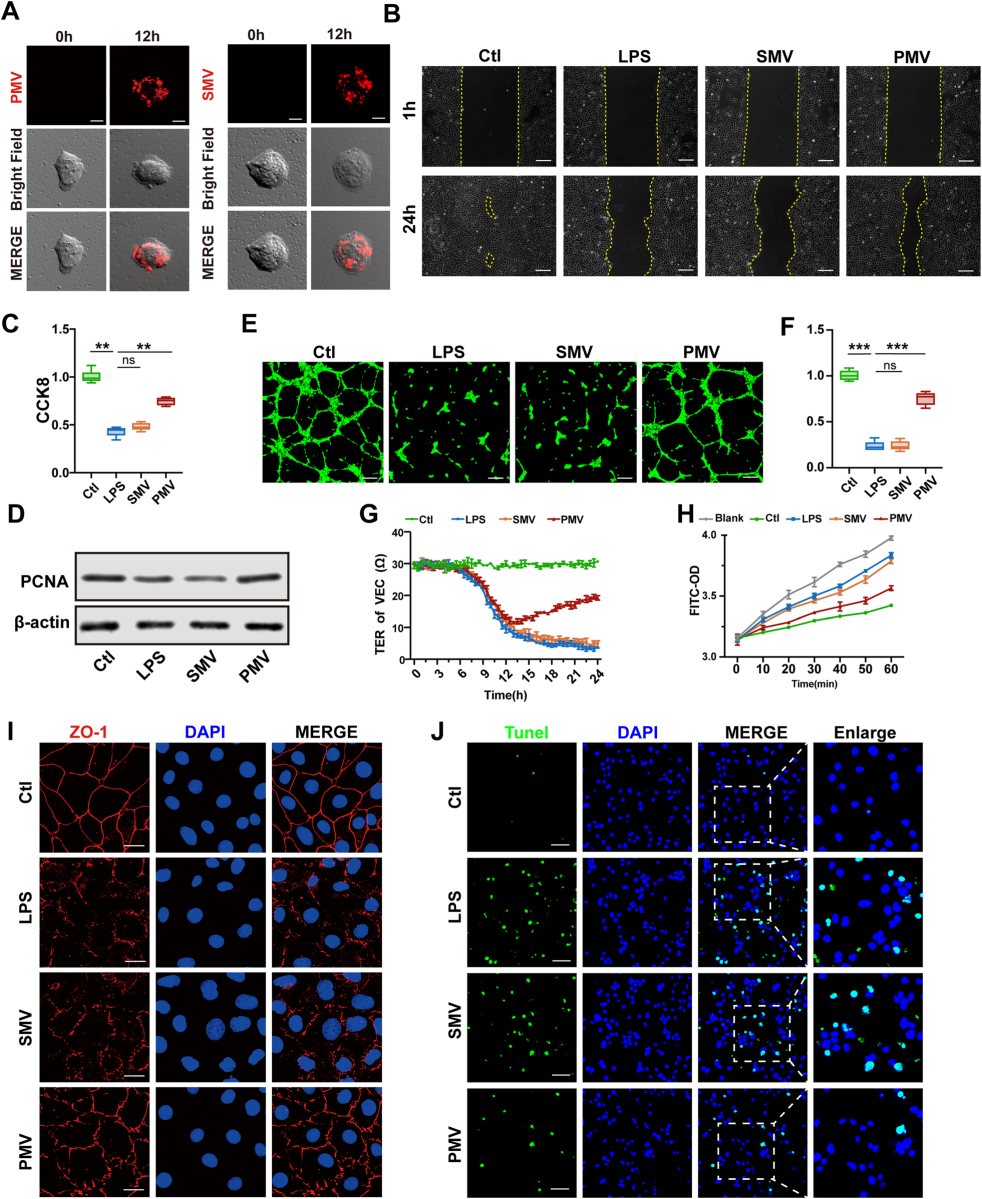
Protective effect of PMVs on LPS-stimulated VECs. **A** Laser confocal microscopy analysis of the absorption of PKH-26 labeled PMVs and SMVs in VECs, the representative microimages are shown (n = 3, bar = 10 μm). **B** Scratch experiment of VECs, effect of PMVs on migration ability is shown (n = 5, bar = 100 μm). **C** CCK8 proliferation of VECs (n = 6). **D** The expression of PCNA in VECs (n = 3). **E** Tube formation of VECs, the representative images are shown (n = 5, bar = 50 μm). **F** Statistical data of the tube length in (E) (n = 5). **G** Transendothelial electrical resistance (TER) of VECs (n = 3). **H** FITC-BSA leakage in VECs (n = 3). **I** Laser confocal microscopy analysis of ZO-1 in VECs, the representative images are shown (n = 5, bar = 20 μm). **J** Laser confocal microscopy analysis of Tunel assay in VECs, the representative images are shown (n = 5, bar = 50 μm). Ctl: normal group; LPS: LPS group; SMV: SMV-treated LPS group; PMV: PMV-treated LPS group. ***P < 0.001; **P < 0.01

To further verify the therapeutic effects of PMVs in vitro, PMVs and SMVs were labeled with red fluorescent dye PKH-26 and further co-incubated with VECs. The results showed that PMVs were incorporated into VECs in a time-dependent manner by laser confocal microscopy (Fig. 3A). Based on the above results, PMVs and SMVs were co-incubated with LPS-stimulated VECs (1 × 10^6^ MVs/mL, at 1 h after LPS stimulation). The results showed that the migration ability of VECs was significantly decreased after LPS stimulation, SMVs had no effect on VECs migration, while PMVs increased the migration ability (Fig. 3B). CCK8 results showed that LPS stimulation decreased the proliferation ability of VECs, and SMVs had no effect on the ability of proliferation, while PMVs restored the proliferation ability significantly (*p* < 0.05) (Fig. 3C). Western blotting of PCNA proved the protective effects of PMVs on cell proliferation after LPS stimulation as well (Fig. 3D). Confocal microscopy results showed that the tube formation capacity of VECs was significantly decreased after LPS stimulation, manifested as the decrease of vessel loops and total length of tubes, while PMVs improved the tube formation capacity significantly (Fig. 3E, F). Meanwhile, LPS stimulation increased the endothelial leakage, manifested as the decrease of TER and the increase of FITC-BSA leakage, and destruction of ZO-1. The administration of PMVs could decrease TER and BSA leakage and improve ZO-1 expression, thus alleviated endothelial leakage (Fig. 3G–I). In addition, a significant decrease in apoptotic VECs was observed after PMV administration (Fig. 3J). The above results suggested that PMVs could improve the function of VECs in sepsis.

### Proteomic analysis in PMVs

**Fig. 4:**
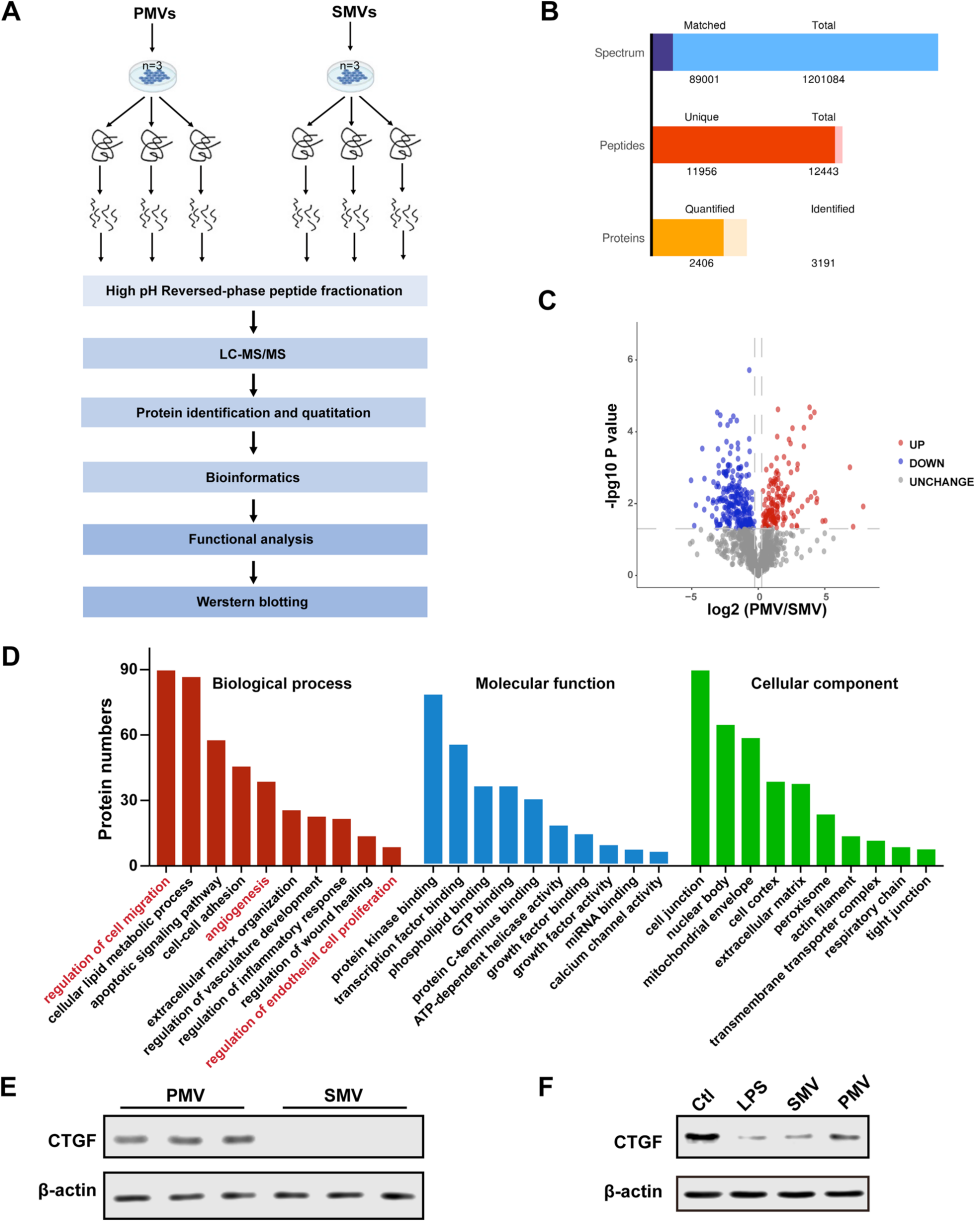
Proteomic analysis of PMVs. **A** Basic schematic diagram of workflow of proteomic analysis of PMVs and SMVs. **B** Proteomic information of spectrum, peptides, and proteins in MVs. **C** Volcano plot analysis of differentially expressed (DE) proteins in MVs (a cutoff of absolute fold change > 1.5 and *p* value < 0.05). **D** GO enrichment analysis is used to classify the DE proteins in MVs, and strategies of biological process, molecular function, and cell component are performed. **E** The expression of CTGF in PMVs and SMVs (n = 3). **F** The effects of PMVs on the expression of CTGF in VECs (n = 3)

To explore the protective mechanism of PMVs, we performed proteomic analysis to measure the protein expression profile in PMVs and SMVs by mass spectrometry (Fig. 4A). A total of 3191 proteins were identified in two types of MVs, among which 2406 proteins contained quantitative information (Fig. 4B). A fold change > 1.5 and *P* value < 0.05 was considered as differentially expressed (DE) protein, and all DE proteins were listed on the volcano plots of the two types of MVs (Fig. 4C), and 129 proteins were upregulated, and 225 proteins were downregulated. Gene ontology (GO) enrichment analysis was conducted to classify the DE proteins in three strategies, including cell component, molecular function, and biological process, among which angiogenesis and cell proliferation had been focused on (Fig. 4D). The data showed that, significantly different proteins relating to angiogenesis and cell proliferation included PRKCD, RPTO, AIMP1, ANGPT1, CTGF and PTN.

Among the identified DE and related biological processes, we focused on the connective tissue growth factor (CTGF), a protein closely involved in angiogenesis, as it had been proven to be associated with several biological functions such as fibrosis, cell adhesion, migration, and tissue remodeling [42, 43]. Our results of Western Blot showed that CTGF expression was significantly higher in PMVs than in SMVs (Fig. 4E), which was consistent with the proteomic analysis. Meanwhile, as displayed in Fig. 4F, it was found that the administration of PMVs significantly improved the expression of CTGF in LPS-stimulated VECs, while SMVs did not effectively change the decrease of CTGF. Therefore, we hypothesized that PMVs exert the protective role on VECs as a result of delivered CTGF.

### PMVs-delivered CTGF mediated the protective effects on the function of VECs

**Fig. 5:**
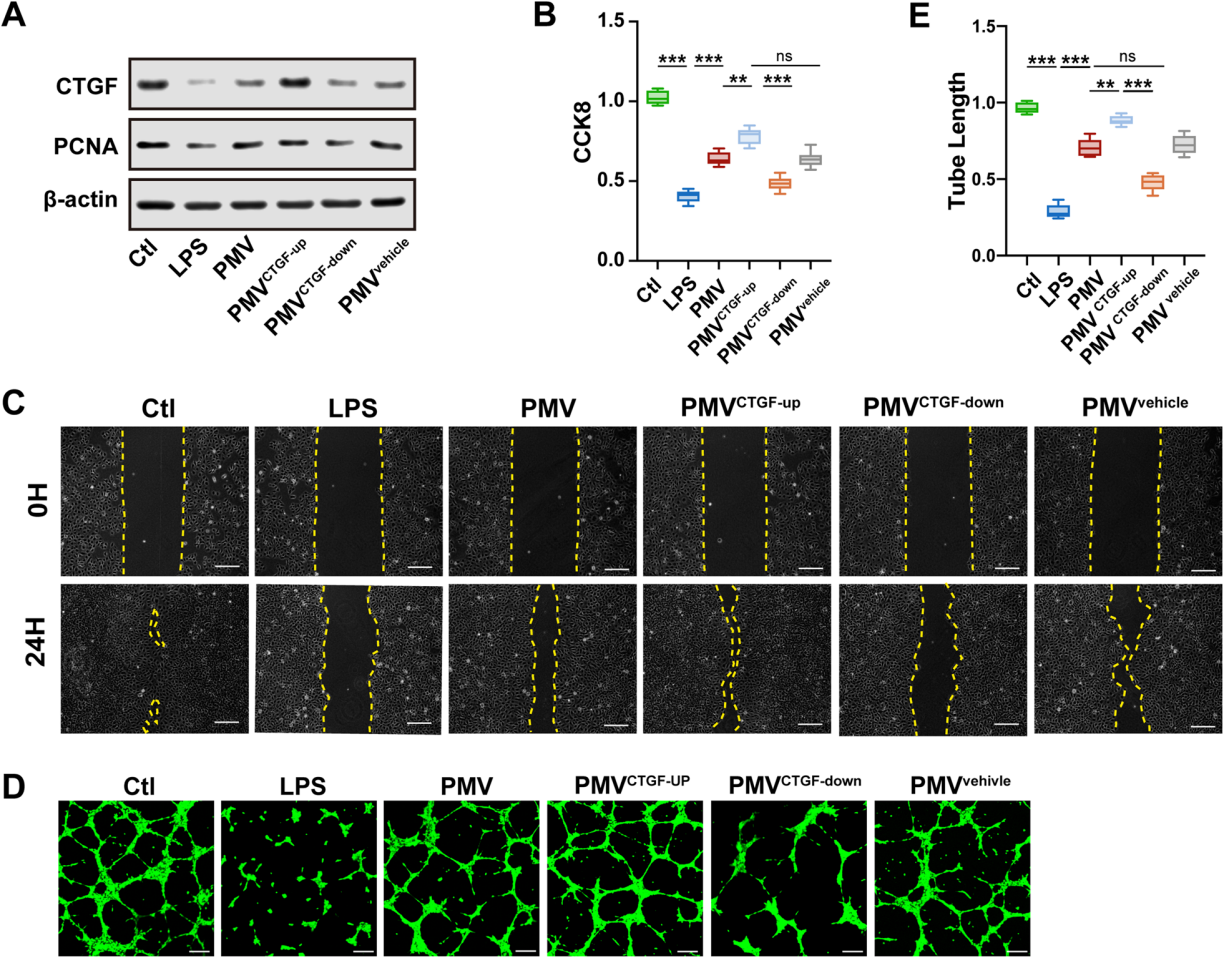
PMVs improved the function of VEC via CTGF delivery. **A** The expression of CTGF and PCNA in LPS-stimulated VECs treated by modified PMVs (n = 3). **B** CCK8 proliferation of LPS-stimulated VECs treated by modified PMVs (n = 6). **C** Scratch experiment of LPS-stimulated VECs treated by modified PMVs, the representative images are shown (n = 5, bar = 100 μm). **D** Tube formation of LPS-stimulated VECs treated by modified PMVs, the representative images are shown (n = 5, bar = 50 μm). **E** Statistical analysis of the tube length in **D** (n = 5). Ctl: normal group; LPS: LPS group; PMV: PMV-treated LPS group; PMV^CTGF−up^: CTGF-upregulated PMV + LPS group; PMV^CTGF−down^: CTGF-downregulated PMV + LPS group; PMV^vehicle^: CTGF-mock-transfected PMV + LPS group. ***P < 0.001, **P < 0.01

To investigate whether CTGF in PMVs participated in the improvement of cellular functions, CTGF-overexpressing adenovirus, CTGF-shRNA adenovirus, and CTGF mock adenovirus were used to transfect PCs, then modified PMVs (PMV^CTGF−up^, PMV^CTGF−down^, PMV^vehicle^) were harvested and further co-incubated with VECs in the presence of LPS. The results indicated that the expression of CTGF in VECs was higher in PMV^CTGF−up^ group compared with PMVs group (Fig. 5A), and the expression of CTGF in PMV^CTGF−down^ group was decreased, which was a little higher than LPS group, suggesting that the overexpression of CTGF in VECs mainly resulted from the PMV-carried CTGF. Meanwhile, compared with PMVs group, PMV^CTGF−up^ further increased the expression of PCNA, while low expression of CTGF in PMVs reduced the expression of PCNA in VECs, and the vehicle-transfected PMVs showed no statistical difference, suggesting that PMVs may promote the proliferation of VECs through CTGF-regulated PCNA expression. Compared with PMVs group, the proliferation and migration ability of VECs was further improved in PMV^CTGF−up^ group, while the proliferation and migration ability of VECs was decreased in PMV^CTGF−down^ group, and there was no statistical difference between PMV^vehicle^ group and PMVs group (Fig. 5B, C). Besides, upregulation of CTGF in PMVs further enhanced endothelial cell tube formation as well (Fig. 5D, E). Collectively, these data showed that PMVs delivered CTGF to VECs to promote cell proliferation and angiogenesis, and subsequently improved cellular function.

### The protective effects of PMV-delivered CTGF depended on the phosphorylation of ERK1/2

**Fig. 6:**
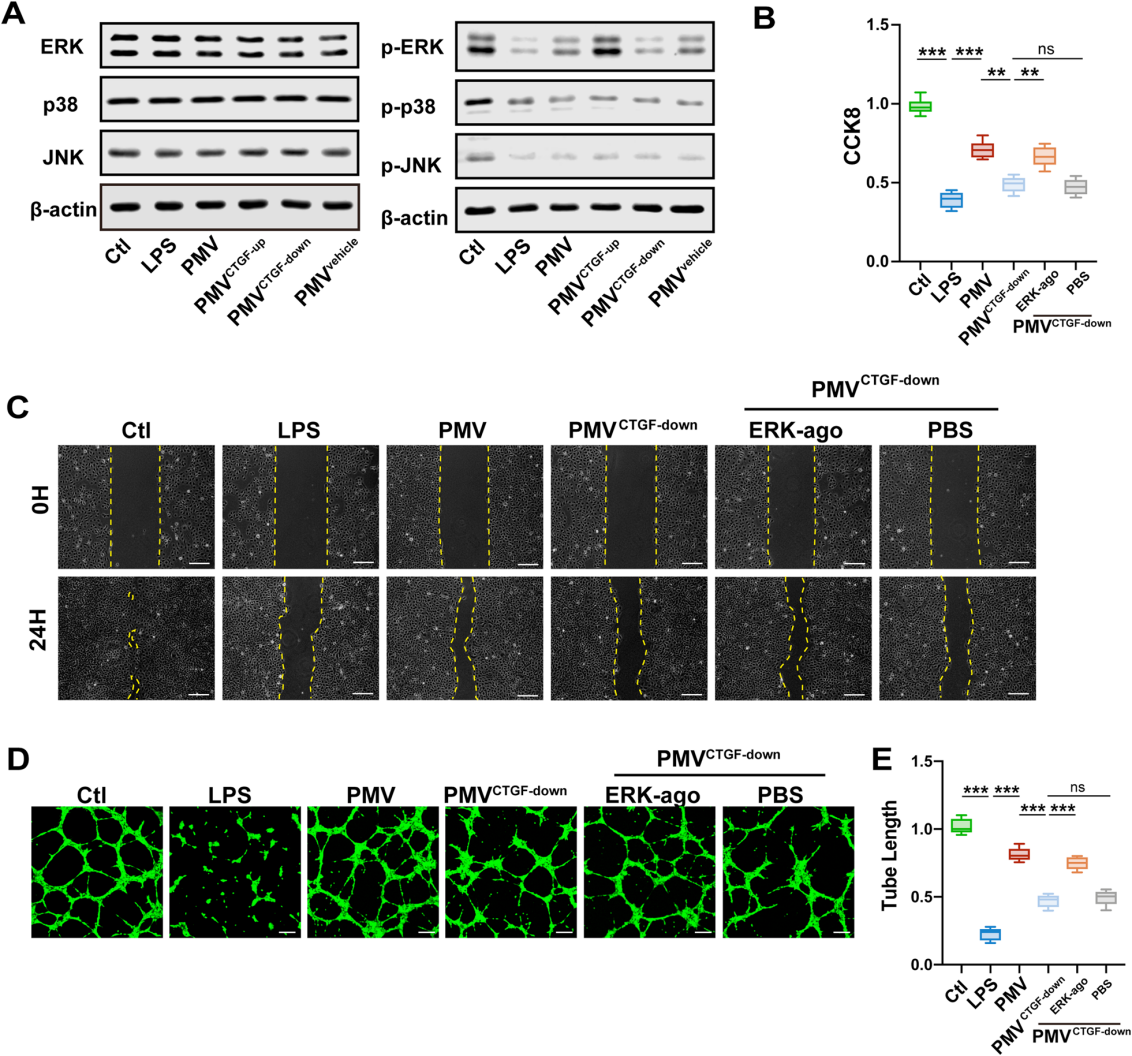
PMV-delivered CTGF improved the function of VEC by activating ERK1/2. **A** The expression and the phosphorylation level of ERK1/2, p38 MAPK, and JNK in LPS-stimulated VECs treated by modified PMVs (n = 3). **B** CCK8 proliferation of LPS-stimulated VECs treated by modified PMVs with or without ERK1/2 agonist (n = 6). **C** Scratch experiment of LPS-stimulated VECs treated by modified PMVs with or without ERK1/2 agonist, the representative images are shown (n = 5, bar = 100 μm). **D** Tube formation of LPS-stimulated VECs treated by modified PMVs with or without ERK1/2 agonist, the representative images are shown (n = 5, bar = 50 μm). (E) Statistical analysis of the tube length in **D** (n = 5). Ctl: normal group; LPS: LPS group; PMV: PMV-treated LPS group; PMV^CTGF−down^: CTGF-downregulated PMV + LPS group. ERK-ago: ERK1/2 agonist Senkyunolide I. ***P < 0.001, **P < 0.01

Previous studies demonstrated that the biological effects of CTGF were mediated by activation of MAPK signaling pathway in different types of cells [44, 45]. The MAPK signaling pathway plays a crucial role in cell proliferation and migration, containing three important subfamilies, ERK1/2, p38 MAPK, and JNK, respectively [44–47]. Thus, we examined the levels of ERK 1/2, p38 MAPK, and JNK proteins in VECs treated with LPS and modified PMVs. The results confirmed that the total protein of ERK1/2, p38 MAPK, and JNK were unchanged, while their phosphorylation levels appeared various degree of descending after LPS stimulation; whereas if co-incubated with PMVs, the phosphorylation level of ERK1/2 (p-ERK1/2) was significantly upregulated compared to p-p38 MAPK and p-JNK. The overexpression of CTGF in PMVs further increased the p-ERK1/2 level, while low expression of CTGF in PMV inhibited the p-ERK1/2 level, suggesting that PMVs may play the protective effect possibly through activation of ERK1/2 (Fig. 6A).

To determine whether ERK1/2 pathway is involved in mediating CTGF-induced protection, PMV^CTGF−down^ were co-incubated with VECs at the presence of ERK1/2 specific agonist Senkyunolide I (SENI, 5 μM). The results showed that the therapeutic effects of PMV^CTGF−down^ on proliferation and migration ability of VECs were significantly decreased compared with PMVs group, EKR1/2 agonist recovered the beneficial effect of PMV^CTGF−down^, indicating that therapeutic effects of PMVs was highly related to the activation of ERK1/2 (Fig. 6B,C). The results of angiogenesis were consistent with the data mentioned before, as ERK1/2 agonist recovered the protective effect of PMV^CTGF−down^ on tube formation capacity (Fig. 6D, E). The results indicated that PMV-delivered CTGF improved the function of VECs by activating ERK1/2.

### PMVs protected functions of VECs by activating ERK1/2-STAT3 axis

**Fig. 7:**
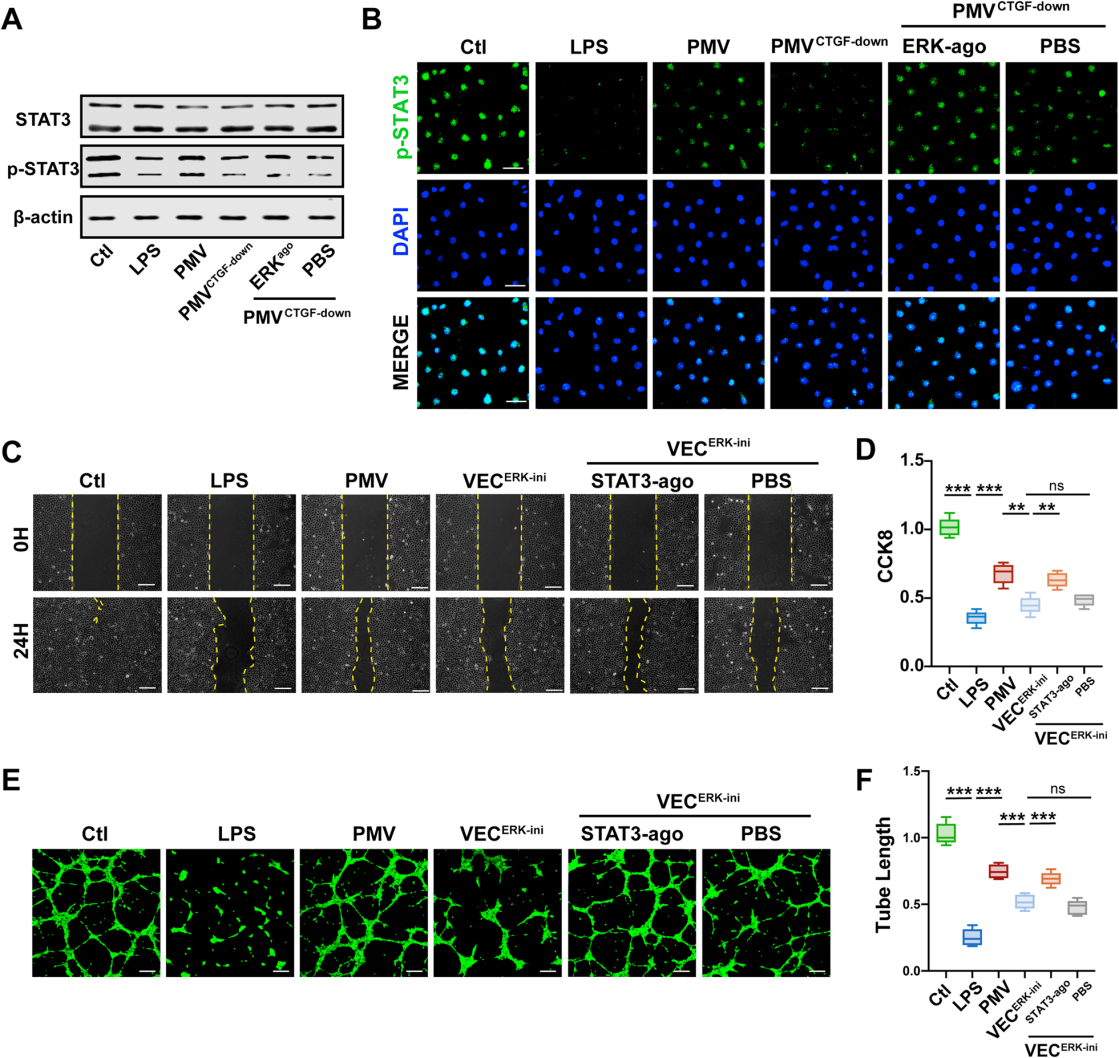
The therapeutic effect of PMV-delivered CTGF was mediated by ERK1/2-STAT3 axis. **A** The expression and the phosphorylation level of STAT3 in LPS-stimulated VECs treated by modified PMVs with or without ERK1/2 agonist (n = 3). **B** Laser confocal microscopy analysis of p-STAT3 (green) in LPS-stimulated VECs treated by modified PMVs with or without ERK1/2 agonist (n = 5, bar = 40 μm). **C** Scratch experiment of LPS-stimulated VECs treated by ERK1/2 inhibitor with or without STAT3 agonist, the representative images are shown (n = 5, bar = 100 μm). **D** CCK8 proliferation of LPS-stimulated VECs treated by ERK1/2 inhibitor with or without STAT3 agonist (n = 6). **E** Tube formation of LPS-stimulated VECs treated by ERK1/2 inhibitor with or without STAT3 agonist, the representative images are shown (n = 5, bar = 50 μm). **F** Statistical analysis of the tube length in **E** (n = 5). Ctl: normal group; LPS: LPS group; PMV: PMV-treated LPS group; PMV^CTGF−down^: CTGF-downregulated PMV + LPS group. ERK-ago: ERK1/2 agonist Senkyunolide I. ERK-ihi: EKR1/2 inhibitor SCH772984. STAT3-ago: STAT3 agonist UVB. ***P < 0.001, **P < 0.01

Considering that the ERK1/2 signaling pathway usually regulates STAT3 expression through activation of its phosphorylation [45, 46, 48], so we detected the total protein and its phosphorylation level of STAT3 (p-STAT3) after co-incubated with PMVs and ERK1/2 agonist. As we expected, PMVs increased the phosphorylation level of STAT3 obviously, while low expression of CTGF in PMVs decreased the phosphorylation level and the presence of ERK agonist increased p-STAT3, indicating that the increase of p-STAT3 resulted from EKR1/2 (Fig. 7A).

Meanwhile, PMVs could increase the expression of p-STAT3 in cell nucleus, which indicated that the activation of p-STAT3 might promote the target gene transcription in nucleus (Fig. 7B). Further results showed that EKR1/2 inhibitor (SCH772984, SCH, 5 μM) may reverse the therapeutic effect of PMVs on the proliferation, migration, and angiogenesis abilities of VECs, while STAT3 agonist (Colivelin TFA, 50 μg/mL) could recover the therapeutic effect of PMVs, suggesting that the protective role of PMVs was mediated by the activation of ERK1/2-STAT3 axis (Fig. 7C–F).

### PMV-delivered CD44 mediated the absorption of PMVs into VECs

**Fig. 8:**
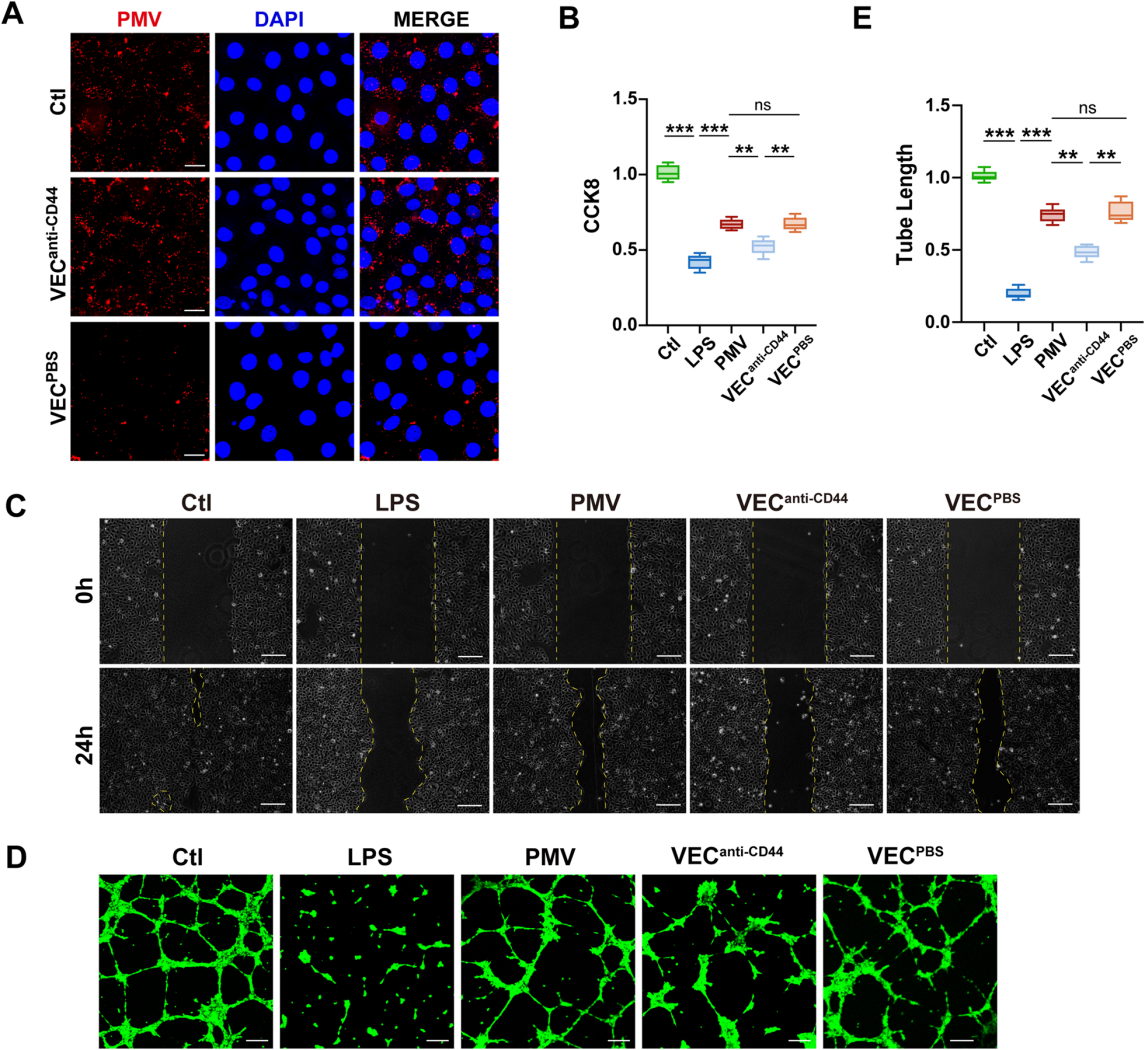
PMV-delivered CD44 mediated the absorption of PMVs. **A** Laser confocal microscopy analysis of the absorption of VECs to PKH-26 labeled PMVs (n = 3, bar = 20 μm). **B** CCK8 proliferation of LPS-stimulated VECs treated by PMVs with anti-CD44 antibody (n = 6). **C** Scratch experiment of LPS-stimulated VECs treated by PMVs with anti-CD44 antibody, the representative images are shown (n = 5, bar = 100 μm). **D** Tube formation of LPS-stimulated VECs treated by PMVs with anti-CD44 antibody, the representative images are shown (n = 5, bar = 50 μm). **E** Statistical analysis of the tube length in **D** (n = 5). Ctl: normal group; LPS: LPS group; PMV: PMV-treated LPS group. Anti-CD44: Anti-CD44 blocking antibody (abcam, America). ***P < 0.001, **P < 0.01

Taken together, the results demonstrate that PMVs improved the proliferation and angiogenesis ability of VECs via CTGF delivery, and then we explored how these PMVs were absorbed by recipient cells. Proteomic results showed abundant CD44 in PMVs, suggesting that CD44 may play an important role in therapeutic effects of PMVs. It has been proved that the uptake of MSC-MVs was mediated by CD44 receptors, which was critical for their beneficial effect [35, 49]. Confocal results indicated that incubation with anti-CD44 blocking antibody decreased the uptake of fluorescent-labeled PMVs into VECs (Fig. 8A). Meanwhile, anti-CD44 significantly reduced the protective effects of PMVs on proliferation, migration and cell tube formation, demonstrating the critical role of CD44 in the uptake of the PMVs into target cells for their therapeutic effects (Fig. 8B–E).

### PMV-delivered CTGF improved pulmonary function in sepsis

**Fig. 9:**
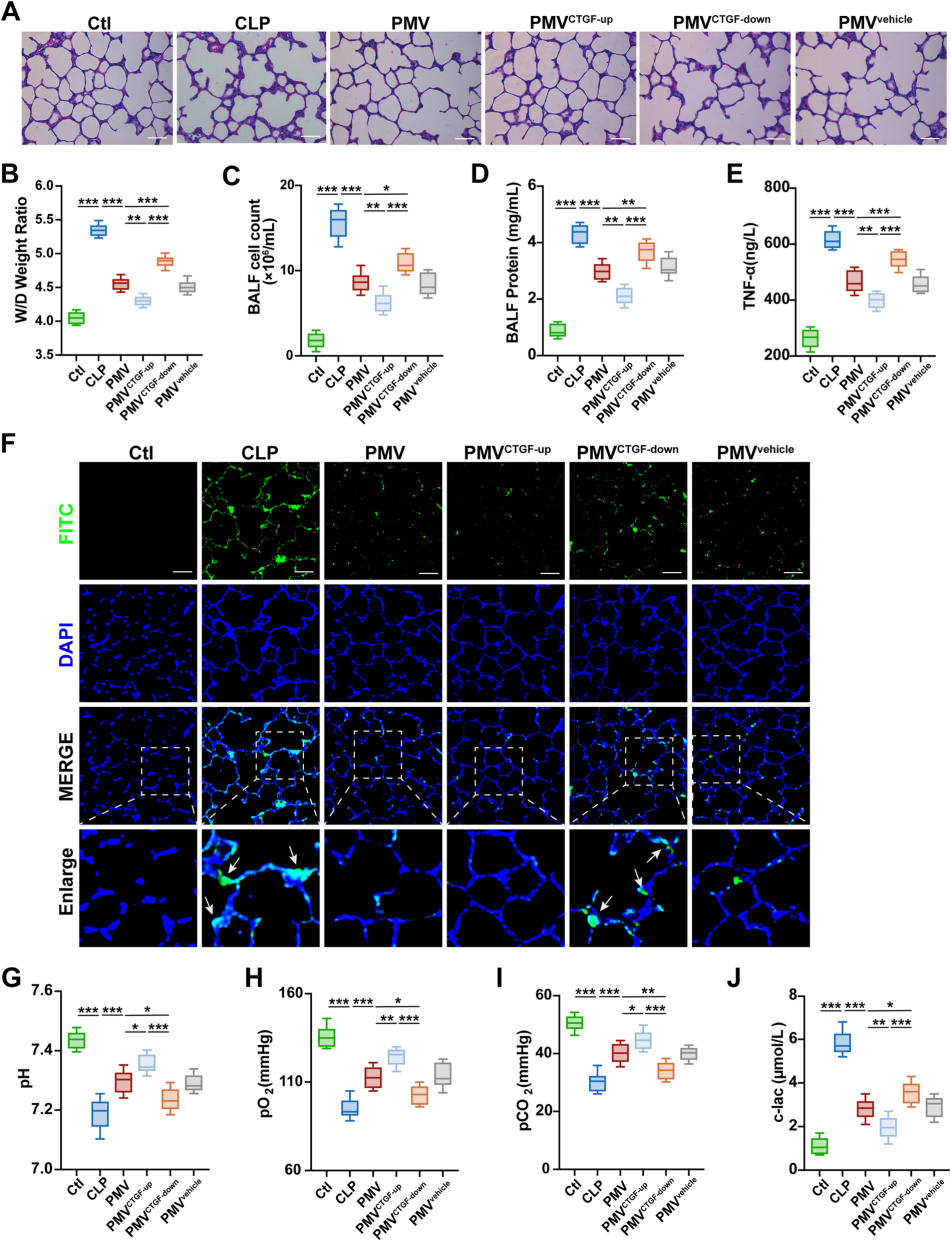
PMVs improved pulmonary function via CTGF delivery. **A** HE staining of lung tissue treated by modified PMVs, the representative images are shown (n = 5, bar = 40 μm). **B** Wet weight to Dry weight ratio of lung in septic rats treated by modified PMVs (n = 8). **C** Cell count in BALF from septic rats treated by modified PMVs (n = 8). **D** Protein concentration in BALF from septic rats treated by modified PMVs (n = 8). **E** Serum TNF-α level in rats (n = 8). **F** Laser confocal microscopy analysis of FITC-BSA leakage in lung tissue treated by modified PMVs, the representative images are shown (n = 5, bar = 50 μm). **G**–**J** Blood gas analysis, including pH, pO_2_, pCO_2_, cLac (n = 8). Ctl: normal group; CLP: sepsis group; PMV: PMV-treated sepsis group; PMV^CTGF−up^: CTGF-upregulated PMV + sepsis group; PMV^CTGF−down^: CTGF-downregulated PMV + sepsis group; PMV^vehicle^: CTGF-mock-transfected PMV + sepsis group. ***P < 0.001, **P < 0.01, *P < 0.05

In order to study the protective effect of PMVs in sepsis, we further injected PMVs, PMV^CTGF−up^, and PMV^CTGF−down^ into septic rats, and the effect on pulmonary function was measured. Compared with CLP group, PMVs decreased the lung tissue damage, FITC-BSA leakage, W/D ratio and BAL protein levels (Fig. 9A); CTGF upregulation in PMVs further increased the protective effects of PMVs, while CTGF downregulation in PMVs inhibited the beneficial effects of PMVs (Fig. 9B–E). These results indicated that PMVs could ameliorate lung injury and improve pulmonary function in septic rats via CTGF delivery.

## Discussion

**Fig. 10:**
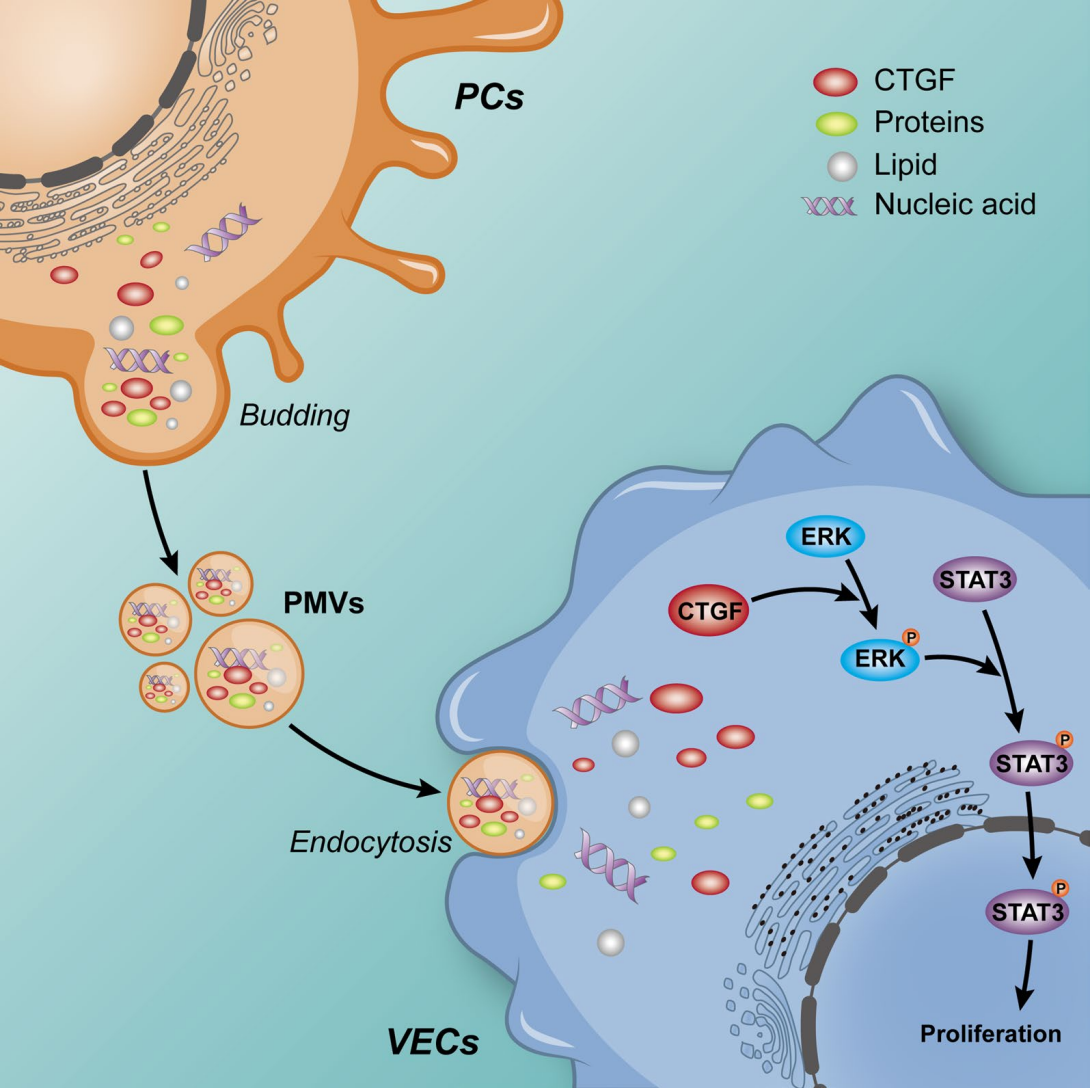
The schematic diagram for the mechanism of the therapeutic effect of PMVs on VEC functions in sepsis. PMVs delivered CTGF to VECs and activating the CTGF-ERK1/2-STAT3 axis, and subsequently improved the proliferation and angiogenesis ability in VECs, thereby restoring vascular functions in sepsis

The present study showed that PMV improved VECs function and pulmonary function after sepsis. By proteomics and GO analysis, we further demonstrated that PMVs delivered CTGF to VECs and improved the proliferation and angiogenesis ability of VECs by activating the ERK1/2-STAT3 signaling pathway to restore the vascular endothelial function (Fig. 10). This study provides a new sight for the treatment of vascular function in sepsis.

Sepsis is a prevalent and severe disease in clinic with a high incidence and mortality rate, which is characterized by the body’s immune disorder to infection, while the treatment strategies are still limited [1–3]. There are numerous toxins and inflammatory factors in the circulation after sepsis, such as LPS, TNF-α, IL-1β, and IL-6, etc., which directly interfere with cell survival, proliferation ability, and barrier function in VECs, and this leads to endothelial leakage, apoptosis, even cell death, and further aggravates the organ dysfunction after sepsis [8–10, 50, 51]. Angiogenesis is an important physiological process that regulates endothelial homeostasis and plays an important role in maintaining vascular function, which is regulated by amount of growth factors [52]. During sepsis, the expression of VEGF, Tie-2, and Ang-1 in VECs are decreased, which leads to the insufficiency of proliferation ability and the dysfunction of VECs [6, 52]. The current treatments on vascular function in sepsis are mainly conventional strategies such as vasopressors and glucocorticoid, so it is urgent to look for effective treatment [5, 6, 8]. In the present study we confirmed that the proliferation and angiogenesis ability of VECs were impaired after sepsis, and PMVs could improve the function of VECs significantly.

PCs are multipotent parietal cells distributed on the periphery of capillaries or microvessels, and they can interact with VECs by direct contact and paracrine effect, and subsequently regulate the function of VECs [12, 53]. It has been proved that PCs could promote vascular contraction by activating PDGFβ and Ang-1 signaling, and improve endothelial permeability and endothelial vascularization [54–56]. According to present studies, retina of weanling SD rats were used to obtain PCs, as the retina of weanling SD rats contained more PCs and the cell proliferation ability was higher than old rats [28].

Recently, studies revealed that PCs could secrete microvesicles to regulate the function of target cells [15]. Microvesicles are membrane vesicles with a diameter range of 100–1000 nm, carrying a lot of bioactive substances as proteins, lipids, and nucleic acids, which play important roles in many diseases [18–20]. Studies indicated that stem cell-derived microvesicles had many advantages due to the low immunogenicity and high targeting ability, thus we speculated that PMVs could improve endothelial function in sepsis [57]. Our study confirmed that PCs could secrete PMVs carrying surface marker of PCs, to promote the proliferation, migration, and angiogenesis ability of VECs, and reduce the pulmonary vascular leakage and improve lung function.

In order to explore the therapeutic mechanism of PMVs on vascular endothelial function, we performed the proteomic analysis for PMVs by mass spectrometry. The results showed that a total of 2406 proteins were identified, and 129 proteins were highly expressed in PMVs. Further GO enrichment and BP analysis found that proangiogenic protein CTGF was highly expressed in PMVs. Research showed that CTGF is known as connective tissue growth factor or CCN2, which belongs to CCN family [42, 43]. The CCN family is a family of extracellular matrix regulatory proteins, and they can participate in the regulation of multiple signal pathways such as Wnt and AMPK signaling, and thus play important roles in cell proliferation, differentiation, adhesion, and development [42, 58, 59]. Several studies showed that stem cells could directly secrete CTGF into circulation to regulate the target cells, while the secreted-CTGF was not stable enough because of the proteases and macrophages in circulation [60, 61], while PMVs could provide shelters for proteins. Our present study found that PMVs could deliver CTGF to activate ERK1/2-STAT3 axis in VEC, thus promote the early stage of angiogenesis. Several study proved that CTGF could regulate the activity of angiogenetic factors including VEGF, ANG-2, MMPS, etc., which could subsequently regulate the stability of ECM [42, 43]. As a result, whether CTGF may play a role in other stages of angiogenesis by different mechanisms still needs further study. Furthermore, there are many other proteins highly expressed in PMVs according to the proteomic results, whether or not they could take part in the regulation of VECs needs to be further studied.

Studies indicated that the role of CTGF might be related to MAPK family, which is a classic signaling pathway in cells, and plays important roles in regulation of proliferation, differentiation, migration, and apoptosis [44, 45]. The MAPK family mainly includes ERK1/2, p38 MAPK, and JNK, which can regulate gene transcription, catalyze protein kinase or protein phosphorylation once activated [44, 45]. The activated MAPK pathway may be different under different stimulations or different cells, thus the target proteins and effect may be highly specific [62]. For example, ERK1/2 regulates cell proliferation by regulating the expression of RSK kinase, STAT3, and AP-1, and JNK participates in cell proliferation by regulating the expression of IL-2 [44–46, 63]. ERK1/2 is mainly related to regulation of cell survival, while JNK and p38 MAPK are more likely to be related to cell apoptosis [44]. Our study revealed that the phosphorylation levels of ERK1/2, p38 MAPK, and JNK were decreased after sepsis, and PMVs increased the phosphorylation level of ERK1/2 significantly, while having little effect on p-p38 MAPK and p-JNK, which suggested that the role of PMVs was mainly related to the activation of ERK1/2. Available studies suggested that STAT3 was a target protein of ERK1/2, which is one of the classic transcription factors [47, 48, 63]. STAT3 can enter into cell nucleus and promote the transcription of genes such as cycllinD1 and c-Myc, thus promoting cell proliferation and migration [64, 65]. Our present study showed that PMVs could increase phosphorylation level of STAT3 significantly and promoted the proliferation of VECs, while such effect could be inhibited in the presence of EKR-inhibitor, and recovered by the STAT3-agonist. In conclusion, it is suggested that PMVs could improve the function of VECs by activating CTGF-ERK1/2-STAT3 axis.

Proteomics results suggested that PMVs also carried adhesion molecule CD44, which is regarded as a classical membrane receptor related to adhesion and migration. [66, 67]. It could be interacted with many ligands, such as P-selectin (CD62P), L-selectin (CD62L), E-selectin (CD62E), CD74, Src, Fyn, Lck, and fibrin etc. Recent studies revealed that CD44 might be related to cell interaction and participate in migration of MSCs to extracellular matrix [66, 68]. In addition, CD44 was confirmed to take part in absorption and internalization of MSC-derived MVs (MMVs), and anti-CD44 antibody could inhibit the effect of MMVs [35, 49, 69], whether or not CD44 was meaningful in the effect of PMVs was not known. As there were large amount of CD44-ligand on the surface of VEC membrane, VECs were able to interact with PMVs and promote the therapeutic effects. In this study, we confirmed that the internalization of PMVs and the therapeutic effects of PMVs on VEC function were significantly reduced by anti-CD44 blocking antibody, indicating that protective effects of PMVs might be related to CD44. However, VECs could also express CD44 and interact with ligands including aslectins and hyaluronic acid to regulate cellular functions. According to our proteomic data, PMVs didn’t carry selectins, CD74, Src, Fyn, Lck, and fibrin, whether PMVs could interact with CD44 in VECs by other ligands needs further investigation.

Nevertheless, there are still some limitations in our study. First, we only focused on the proteins related to cell proliferation and angiogenesis in PMVs, whether or not other proteins have the same protective effect on VECs functions needs to be further studied. Next, the present study confirmed that PMVs protect vascular endothelial function in sepsis, whether PMVs have protective effect on other process, such as apoptosis and autophagy remains to be intensively studied.

## Conclusions

PMVs have a protective effect on vascular endothelial function, thereby ameliorating pulmonary function in sepsis (Fig. 10). The mechanism is related to the improvement of proliferation and angiogenesis ability in VECs by activating CTGF-ERK1/2-STAT3 axis, thereby restoring vascular functions. The finding provides a new sight for treatments of sepsis.

## Data Availability

All of the data that support the findings of this study are available from the corresponding author Tao Li upon reasonable request.

## Abbreviations

Ago: Agonist
CLP: Cecal ligation and puncture
CTGF: Connective tissue growth factor
FCM: Flow-cytometry
GO: Gene ontology
ini: Inhibitor
KEGG: Kyoto encyclopedia of genes and genomes
LPS: Lipopolysaccharide
MV: Microvesicle
PC: Pericyte
PMV: Pericyte-derived microvesicle
SD: Sprague–Dawley
SMV: Smooth muscle cell-derived microvesicle
TEM: Transmission electron microscopy
TER: Transendothelial electrical resistance
VEC: Vascular endothelial cell
